# Analysis of N6-Methyladenosine Methyltransferase Reveals METTL14 and ZC3H13 as Tumor Suppressor Genes in Breast Cancer

**DOI:** 10.3389/fonc.2020.578963

**Published:** 2020-12-09

**Authors:** Peng-Ju Gong, You-Cheng Shao, Yan Yang, Wen-Jing Song, Xin He, Yi-Fan Zeng, Si-Rui Huang, Lei Wei, Jing-Wei Zhang

**Affiliations:** ^1^Department of Breast and Thyroid Surgery, Zhongnan Hospital, Hubei Key Laboratory of Tumor Biological Behaviors, Hubei Cancer Clinical Study Center, Wuhan University, Wuhan, China; ^2^Department of Pathology and Pathophysiology, Hubei Provincial Key Laboratory of Developmentally Originated Disease, School of Basic Medical Sciences, Wuhan University, Wuhan, China

**Keywords:** m6A, METTL14, ZC3H13, breast cancer, prognosis, APC, Wnt signaling pathway

## Abstract

**Objectives:**

Recently, an increasing number of studies have revealed that N6-methyladenosine (m6A) functions as a significant post-transcriptional modification which plays a critical role in the occurrence and progression of enriched tumors by regulating coding and non-coding RNA biogenesis. However, the biological function of m6A in breast cancer remains largely unclear.

**Materials and Methods:**

In this study, we used a series of bioinformatic databases and tools to jointly analyze the expression of m6A methylation transferases (METTL3, METTL14, WTAP, RBM15, RBM15B and ZC3H13) and investigate the prognostic value of METTL14 and ZC3H13 in breast cancer. Besides, we analyzed the downstream carcinogenic molecular mechanisms related to METTL14 and ZC3H13 and their relationship with immune infiltration in breast tumor tissues.

**Results:**

The results showed that METTL14 and ZC3H13 were the down-regulated m6A methylation transferases in breast cancer. Survival outcome analysis suggested that abnormally low expression of METTL14 and ZC3H13 could predict unfavorable prognosis in four breast cancer subtypes. Moreover, their down-regulation was associated with ER-, PR- and triple-negative breast cancer patients, as well as tumor progression (increased Scarff, Bloom and Richardson grade status and Nottingham Prognostic Index classification). Co-expression analysis revealed that METTL14 and ZC3H13 had a strong positive correlation with APC, an antagonist of the Wnt signaling pathway, indicating they might cooperate in regulating proliferation, invasion, and metastasis of tumor cells. METTL14, ZC3H13, and APC expression levels had significant positive correlation with infiltrating levels of CD4+ T cells, CD8+ T cells, neutrophils, macrophages, and dendritic cells, and negative correlation with Treg cells in breast cancer.

**Conclusions:**

This study demonstrated that down-regulation of METTL14 and ZC3H13 which act as two tumor suppressor genes was found in breast cancer and predicted poor prognosis. Their abnormal expression promoted breast cancer invasion by affecting pathways related to tumor progression and mediating immunosuppression.

## Introduction

The morbidity and mortality of breast cancer rank first and second in global female tumors, respectively. Globally, the incidence of breast cancer has been increasing year by year, and the age of onset has gradually become younger (1). The incidence of breast cancer is relatively low in China, but the number of newly diagnosed breast cancer cases has been increasing continuously in recent years. It has already been the malignant tumor with the highest incidence among women in some large or medium-sized cities, which seriously threatens women’s health and life, and brings huge economic and health problems to the society of China. Although the prognosis of breast cancer continues to improve with the progress of treatment, breast cancer is still the main cause of female death at present (2).

The occurrence and development of tumors are driven by the disorder of genetic, epigenetic, and environmental factors, and epigenetic factors play an important role as a bridge between genetic and environmental factors (3, 4). RNA modifications are also important types of post-transcriptional epigenetic modification, and N6-methyladenosine (m6A) is the most predominant modification of mRNA. In mammalian cells, there is an average of 1–2 m6A sites per 1,000 nucleotides (5, 6). At present, many proteins involved in m6A have been identified, which are classified into m6A methylation transferases (“writer” proteins), m6A demethylases (“eraser” proteins), and m6A reading and binding proteins (“reader” proteins) (7). RNA is methylated at the sixth nitrogen atom of adenylate by the action of “writer” proteins, and methyltransferase-like 3 (METTL3) and methyltransferase-like 14 (METTL14) form a hetero complex, which complete this modification process together with Wilms tumor 1 associated protein (WTAP) and other factors, such as Putative RNA-binding protein 15 (RBM15) and Zinc Finger CCCH-Type Containing 13 (ZC3H13). By contrast, the m6A modified regions can be demethylated under the action of two “eraser” proteins, fat mass, and obesity-associated protein (FTO) and alkB homolog 5 (ALKBH5), making m6A a reversible modification. These m6A modified sites can be recognized by “readers” proteins to regulate RNA metabolism, including translation, splicing, translocation, degradation, and processing (7).

The abnormal expression of m6A “writer” proteins results in abnormal levels of m6A modification in tumor cells, which further abnormalizes the metabolism of tumor-related genes mRNA, thereby promoting the development of a variety of cancers (8, 9). The up-regulated METTL3 in gastric cancer was positively correlated with the poor prognosis of patients, and METTL3 promoted epithelial–mesenchymal transition of gastric cancer, leading to tumor invasion and metastasis (10). WTAP expression was up-regulated in hepatocellular carcinoma; WTAP-mediated m6A modification led to epigenetic silencing of the tumor suppressor gene ETS1, and promoted the progression of hepatocellular carcinoma by regulating the cell cycle (11). The expression of METTL14 was down-regulated in colorectal cancer, which indicated a poor prognosis of patients, and promoted the proliferation and invasion of tumor cells by inhibiting the degradation of the oncogene XIST (12). However, the abnormal expression of m6A “writer” proteins in breast cancer still remains largely unknown, and the gene targets and molecular mechanisms involved downstream also need to be further elucidated.

In this study, we used Oncomine and The Cancer Genome Atlas (TCGA) databases to jointly analyze the expression of m6A “writer” in breast cancer including METTL3, METTL14, WTAP, RBM15, RBM15B, and ZC3H13, indicating that the mRNA expression levels of METTL14 and ZC3H13 were lower in tumor samples. We further combined the data of TIMER, cBioPortal, Kaplan–Meier plotter and other databases to analyze the prognostic value of METTL14 and ZC3H13 in breast cancer, their co-expressed genes and related signaling pathways, as well as the relationship between their expression levels and infiltrating immune cells in tumor tissues. In total, we highlighted the important role of METTL14 and ZC3H13 in breast cancer, provided promising markers for predicting prognosis of patients, and explained the potential molecular mechanism of the two as tumor suppressor genes.

## Materials and Methods

### Gene Expression Data Mining

ONCOMINE gene expression array dataset (13) (https://www.oncomine.org), an online cancer microarray database, was utilized to analyze the transcription levels of different m6A RNA methylation writer genes: METTL3, METTL14, WTAP, RBM15, RBM15B, and ZC3H13 in different cancers. The cut-off of P value was 0.05 and fold change were 2.0, respectively. Then, METTL3, METTL14, RBM15B, ZC3H13, and APC gene expression levels in various cancer types were further analyzed and verified *via* The Tumor IMmune Estimation Resource 2.0 (TIMER 2.0) algorithm database (14) (http://timer.cistrome.org/) based on data from The Cancer Genome Atlas (TCGA). The heatmaps of the selected gene mRNA expression were conducted and visualized by the UCSC Xena (15, 16) (https://xena.ucsc.edu/public), an interactive online visualization of seminal cancer genomic dataset, based on the data from TCGA.

### Cancer Genomic Analysis

The Breast Invasive Carcinoma (TCGA, PanCancer Atlas) dataset was selected for further analyses of METTL14 and ZC3H13 by cBioPortal (17, 18) (https://www.cbioportal.org/). The genomic profiles containing mutations, putative copy-number alterations, mRNA expression and protein expression were visualized. Then, the correlations between mRNA expression levels of METTL14, ZC3H13, and putative copy-number alterations, mutations were analyzed.

### Survival Outcome Analysis

The prognostic values of METTL14, ZC3H13, APC, and selected hub genes in breast cancer samples were assessed by the online tools and database, Kaplan–Meier plotter (19) (http://kmplot.com/analysis/index.php?p=service&cancer=breast). To analyze the overall survival (OS) and progression-free survival (RFS) of breast cancer patients, patient samples were divided into two groups by the best cut-off value by the tool automatically and calculated *via* the Kaplan–Meier analysis and Logrank-P test. Besides, the prognostic roles of METTL14, ZC3H13, and APC in enriched datasets from Gene Expression Omnibus database (GEO) were further analyzed and verified by PrognoScan database (20) (http://dna00.bio.kyutech.ac.jp/PrognoScan/index.html). The prognostic values of METTL14, ZC3H13, APC, and selected hub genes were also estimated with TCGA breast cancer samples by using Kaplan–Meier plotter.

### The Associations of Gene Expression and Clinical Parameters

The METTL14 and ZC3H13 mRNA expression data and the clinical data of breast cancer patients in TCGA were extracted from the UCSC Xena (15, 16). After deleting incomplete cases, the high and low expression groups were divided by the median value of the mRNA expression level and then for further analysis. Besides, the associations of METTL14 and ZC3H13 gene expression and clinical parameters were also evaluated and verified using the Breast Cancer Gene-Expression Miner v4.0 (bc-GenExMiner v4.0) online dataset (21) (http://bcgenex.centregauducheau.fr/BC-GEM/GEM-Accueil.php?js=1), in accordance with the RNA-seq data and clinical data from TCGA and GEO datasets.

### Gene Correlation Analysis

The top 200 co-expressed genes of METTL14 and ZC3H13 were analyzed and obtained from the Breast Invasive Carcinoma (TCGA, PanCancer Atlas) on cBioPortal by Spearman’s Correlation method (17, 18). Then, the co-expressed genes of the two genes were cross-referenced to obtain a cohort of 76 common co-expressed genes. The correlation between APC and METTL14, ZC3H13 were also analyzed on GEPIA (22) (http://gepia.cancer-pku.cn/detail.php) and bc-GenExMiner v4.0 (21) by Spearman’s and Pearson’s correlation methods, respectively.

### Pathway and Gene Ontology Enrichment

Functional enrichment analysis including Gene Ontology (GO) analysis and Kyoto Encyclopedia of Genes and Genomes (KEGG) pathway enrichment was performed based on the WebGestalt website (23) (http://www.webgestalt.org/option.php) to understand the function of the extracted 76 common co-expressed genes. GO analysis can be divided into three categories: biological processes (BPs), cellular components (CCs), and molecular functions (MFs). P <0.05 was considered significant *via* Fisher’s exact test.

### Protein–Protein Interaction Network and Sub-Module Construction

The STRING database (24) was utilized to construct the PPI network of 76 common co-expressed genes with an interaction score above 4.0 and hiding the unconnected genes. Then, ClueGO APP on Cytoscape 3.7.2 (25) was employed to assess the function of the given PPI network, and the most important module and hub genes of the PPI network were selected and constructed by the MCODE APP according to the rules as follows: degree cut-off = 2, node score cut-off = 0.2, Max depth = 100, and k-score = 2.

### Infiltrating Immune Cell Analysis

The correlation of mRNA expression level of METTL14, ZC3H13, APC with the abundance of seven types of infiltrating immune cells (CD4+ T cells, CD8+ T cells, Treg cells, B cells, neutrophils, macrophages, and dendritic cells) and the association between immune infiltrates and somatic CNV of METTL14 and ZC3H13 in breast cancer patients were calculated using The Tumor IMmune Estimation Resource 2.0 (TIMER 2.0) algorithm database (26, 27) (http://timer.cistrome.org/), noting that the abundance of Treg cells was analyzed by the CIBERSORT method. Tumor purity is an important factor affecting the analysis of immune infiltration in tumor samples by genomic methods.

### Tissue Samples and Immunohistochemical Staining

The breast cancer tissue microarrays (F601101) comprising 50 breast tumor tissues and 10 adjacent normal tissues were purchased from Zhong Ke Guang Huang Biotech (http://bioaitech.com). IHC was performed on the tissue microarrays using the UltraSensitive™S-P Methods. Briefly, after the tissue samples were first dewaxed, H_2_O_2_ was used to block the activity of endogenous peroxidase. The primary antibodies for METTL14 and ZC3H13 were incubated with a diluted solution, and then a secondary antibody was added and developed by diaminobenzoquinone (DAB). At last, hematoxylin was used to counterstain the microarrays. Staining index analyzed by two independent investigators was used to compare the expression of METTL14 and ZC3H13 between tumor samples and normal control. Staining intensity was graded as follows: 0 (−, no staining); 1 (+, weak staining, light yellow); 2 (++, moderate staining, yellow brown); 3 (+++, strong staining, brown). Tumor cells’ proportion was scored according to: 0 (no positive tumor cells); 1 (<10% positive tumor cells); 2 (10–25% positive tumor cells); 3 (26–49% positive tumor cells); 4 (≥50% positive tumor cells). The results were given by randomly observing at least 5–10 HPFs and taking the average. Staining index was the product of staining intensity grade and the score of positive tumor cells.

### Statistical Analysis

Students’ *t* test was employed to compare mRNA expression in Oncomine and TIMER 2.0, also to analyze the staining index. ANOVA was used to identify the significance of METTL14 and ZC3H13 mRNA levels in different putative copy-number alterations, mutations groups. The Kaplan–Meier curve and Log-rank P test in Kaplan–Meier (KM) plotter, and Univariable COX in PrognoScan were used to analyze the survival outcomes. The chi-squared test was used to investigate the significance of the correlation of METTL14 and ZC3H13 expression with clinical parameters in different breast cancer groups. Gene expression significant difference between subgroups is assessed by Welch’s test on bc-GenExMiner v4.0. The correlation of gene expression or infiltrating immune cells was evaluated using the Spearman’s and Pearson’s correlation method. Fisher’s exact test was adopted to measure the gene enrichment. P <0.05 was considered statistically significant.

## Results

### Down-Regulation of METTL14 and ZC3H13 mRNA Expression in Breast Cancer

**Figure 1:**
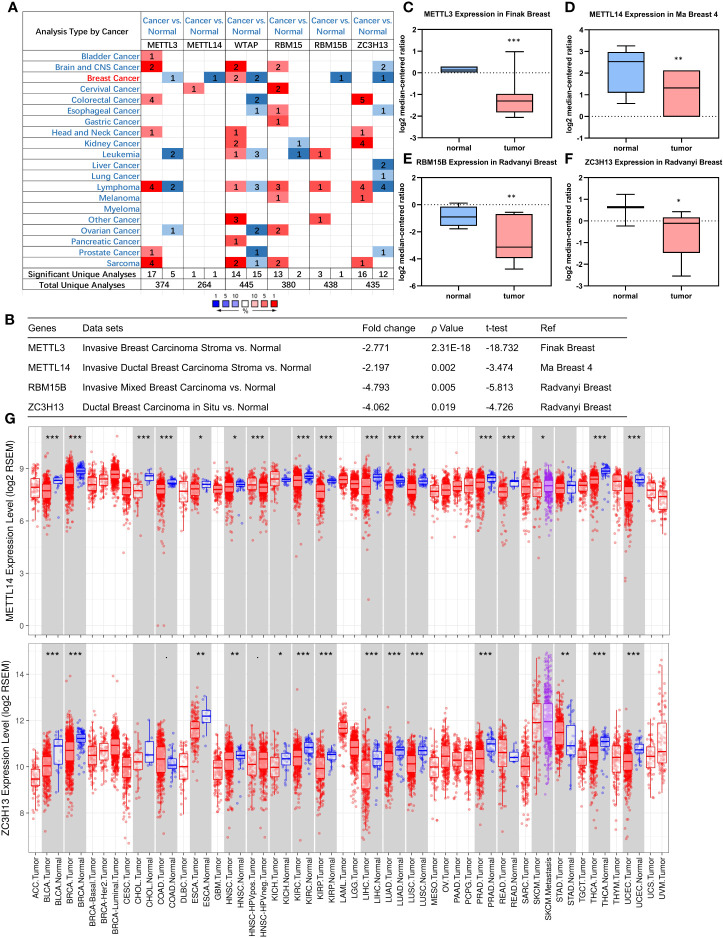
Down-regulation of METTL14 and ZC3H13 mRNA expression in breast cancer. **(A)** Oncomine database indicates the numbers of datasets with statistically significant overexpressed mRNA (red) or downexpressed (blue) of METTL3, METTL14, WTAP, RBM15, RBM15B, and ZC3H13 (cancer tissues *vs* normal tissues). **(B–F)** The expression of METTL3, METTL14, RBM15B, and ZC3H13 in breast cancer tissues *vs* normal control in the datasets of Oncomine database. **(G)** The expression of METTL14 and ZC3H13 in cancer tissues and normal control in the TCGA database generated by TIMER database. ***P < 0.001, **P < 0.01, *P < 0.05.

**Figure 2:**
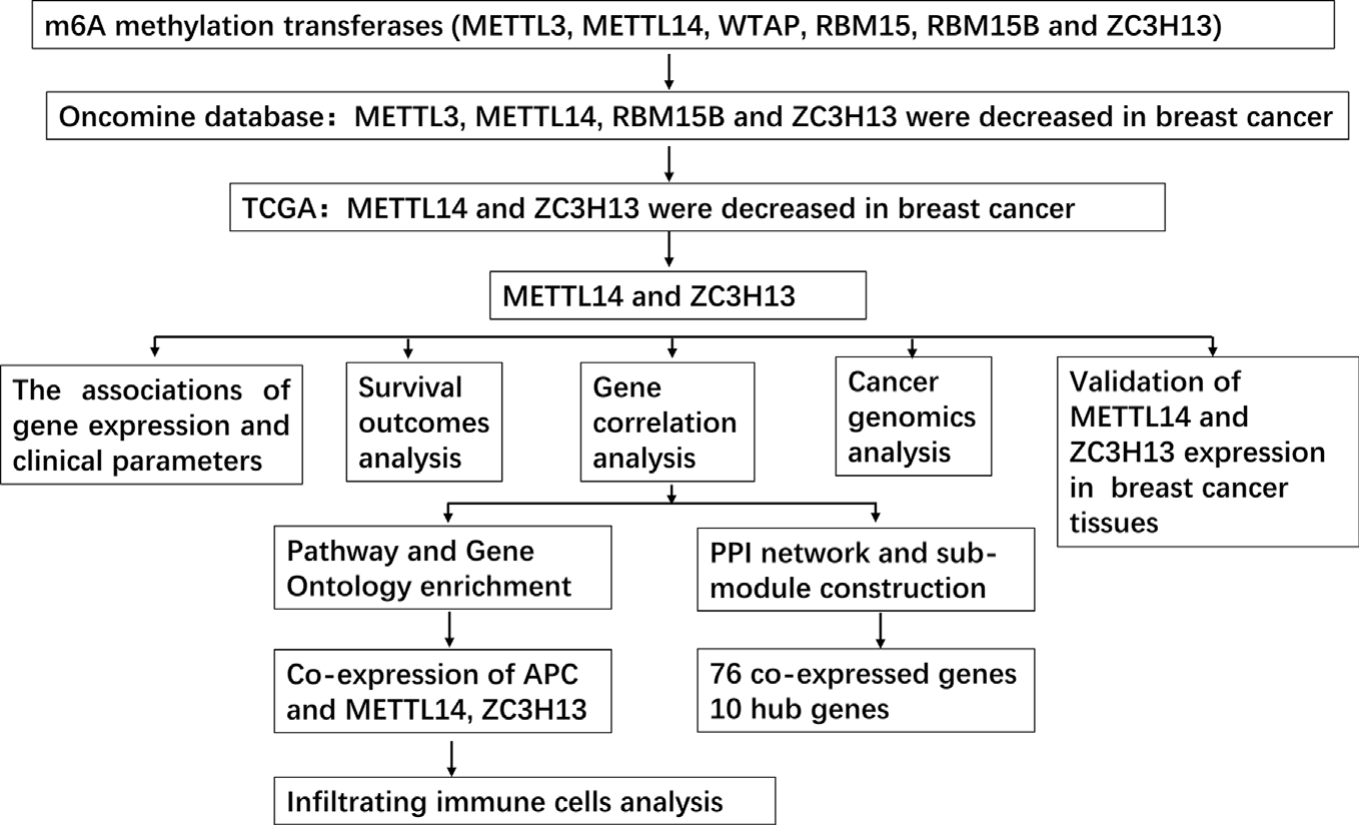
Study flow chart.

First off, the expression profiles of N6-methyladenosine (m6A) RNA methylation associated methyltransferases were analyzed *via* Oncomine database. The expression of METTL3, METTL14, RBM15B, and ZC3H13, but not of WTAP and RBM15, was significantly decreased in breast cancer (**Figure 1A**). More details, Oncomine analysis of cancer *vs* normal samples in enriched breast cancer patient datasets showed that their expression was reduced in invasive breast carcinoma stroma, invasive ductal breast carcinoma stroma, invasive mixed breast carcinoma, and ductal breast carcinoma *in situ*, respectively (**Figures 1B–F**). Furthermore, mining of TIMER database based on TCGA breast cancer samples demonstrated that only the mRNA expression levels of METTL14 and ZC3H13 were also lower in tumor samples (**Figure 1G**), and there was not any difference of METTL3 and RBM15B (**Figure S2**). Considering that WTAP did not carry a consistent gene abnormal expression trend in Oncomine database (**Figures 1A** and **S1**), and that METTL3 and RBM15B were only downregulated in Oncomine database but not in TCGA breast cancer samples (**Figures 1A** and **S2**), we excluded these three genes and only included METL14 and ZC3H13 for subsequent studies (**Figure 2**).

### Rare Genomic Alteration of METTL14 and ZC3H13 in Breast Cancer

**Figure 3:**
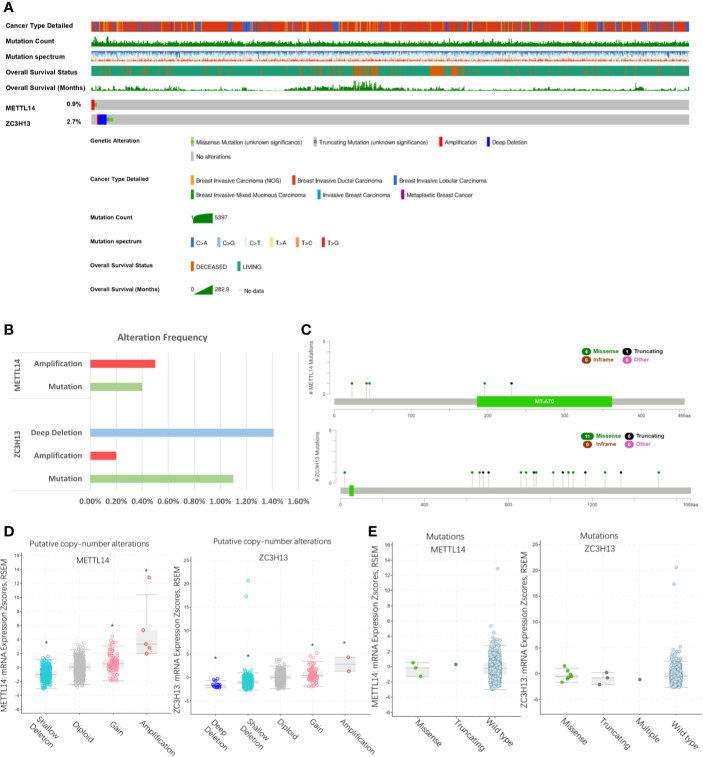
Rare genomic alteration of METTL14 and ZC3H13 in breast cancer. **(A, B)** The genetic alteration rate of METTL14 and ZC3H13 in breast cancer *via* the cBioPortal online database (TCGA, PanCancer Atlas). **(C)** Schematic representation of METTL14 and ZC3H13 mutations using the cBioportal (TCGA, PanCancer Atlas). **(D)** The relationship between expression and putative copy-number alteration from GISTIC of METTL14 and ZC3H13. **(E)** The relationship between mutation of METTL14 and ZC3H13., *P < 0.05.

We evaluated the genomic alteration of METTL14 and ZC3H13 among breast cancer samples by the cBioPortal online database (TCGA, PanCancer Atlas). Overall, about 36 (4%) samples, a very small ratio, had genetic changes found in breast cancer, with 0.9% in METTL14 and 2.7% in ZC3H13, respectively (**Figure 3A**). Moreover, there were 0.5% samples with amplification and 0.4% with mutation in METTL14, and 1.4% with deep deletion, 0.2% with amplification, and 1.1% with mutation in ZC3H13 (**Figure 3B**). The mutation ratio of ZC3H13 was relatively higher, including 11 missense and six truncating points, whereas only four missense mutation points were found in METTL14 (**Figure 3C**). In addition, for the relationship between expression and genomic alteration, the expression of METTL14 and ZC3H13 was much higher in samples with amplification and lower in those with deletion compared with normal samples (**Figure 3D**). However, we did not find any differences between mutant and wild type samples (**Figure 3E**).

### Low METTL14 and ZC3H13 Expression Demonstrate Poor Prognosis in Breast Cancer

**Figure 4:**
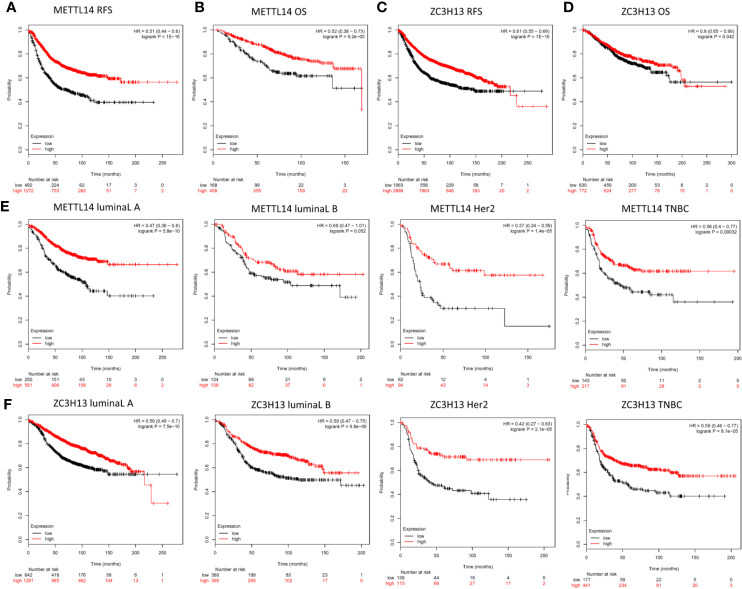
Low METTL14 and ZC3H13 expression demonstrates poor prognosis in breast cancer. **(A–D)** Prognostic significance of METTL14 and ZC3H13 gene expression in breast cancer patients based on the Kaplan–Meier plotter database. RFS, relapse-free survival; OS, overall survival. **(E, F)** The RFS curves of METTL14 and ZC3H13 gene expression in four types breast cancer patients, including luminal type A, luminal type B, HER2 enriched type, and triple negative type (TNBC).

In order to explore the roles of METTL14 and ZC3H13 in the survival of breast cancer patients, the Kaplan–Meier plotter was used to calculate the correlation between the mRNA expression levels and survival. The Kaplan–Meier curve and Log-rank P test confirmed that the low expression of METTL14 (Prob ID: 235552 at) and ZC3H13 (Prob ID: 209851 at) was negatively correlated with the overall survival (OS) and progression-free survival (RFS) (**Figures 4A–D**). But we only found that reduced expression of METTL14 but not ZC3H13 was associated with poor RFS, and there was no correlation between OS and the expression of the two in TCGA breast cancer samples (**Figure S3**). Generally, breast cancer can be divided into four types according to the molecular characteristics, including luminal type A, luminal type B, HER2 enriched type, and triple negative type, which could influence the adjuvant treatments and the prognosis of patients (28). Survival analysis showed that the decreased expression levels of METTL14 and ZC3H13 were related to RFS in all of four types (**Figures 4E, F**). Besides, we also made a validation of their prognostic roles using Prognoscan database and got a consistent trend in various survival types (**Table 1**). Thus, the two genes play a role in tumor-suppressing and can be used as potential prognostic markers of breast cancer.

**Table 1:** Survival analysis of METTL14, ZC3H13 and APC in breast cancer patients (the PrognoScan).

Gene	Dataset	Endpoint	Number	ln(HR-high/HR-low)	COXP-value	ln(HR)	HR [95% CI-low CI-up]
METTL14	GSE1456-GPL97	Relapse Free Survival	159	−0.901843	0.015023	−0.807295	0.45 [0.23–0.86]
	GSE3494-GPL97	Disease Specific Survival	236	−1.03975	0.014308	−1.25269	0.29 [0.10–0.78]
		Disease Specific Survival	236	−0.845651	0.021584	−1.011	0.36 [0.15–0.86]
ZC3H13	GSE9195	Relapse Free Survival	77	-2.4538	0.015768	−1.77061	0.17 [0.04–0.72]
	GSE1456-GPL96	Relapse Free Survival	159	−1.14417	0.04399	−0.971572	0.38 [0.15–0.97]
	GSE1456-GPL97	Overall Survival	159	−1.15878	0.014582	−0.818691	0.44 [0.23–0.85]
		Relapse Free Survival	159	−1.27869	0.009604	−0.858074	0.42 [0.22–0.81]
		Disease Specific Survival	159	−1.61681	0.00257	−1.19064	0.30 [0.14–0.66]
APC	GSE9195	Distant Metastasis Free Survival	77	−1.80092	0.008811	−4.577	0.01 [0.00–0.32]
		Relapse Free Survival	77	−15.5222	0.03119	−3.02081	0.05 [0.00–0.76]
	GSE1379	Relapse Free Survival	60	−2.36113	0.001616	−2.01845	0.13 [0.04–0.47]
	GSE9893	Overall Survival	155	−0.975572	0.015758	−0.351693	0.70 [0.53–0.94]
	GSE1456-GPL96	Disease Specific Survival	159	−1.12282	0.03057	−0.634452	0.53 [0.30–0.94]
		Overall Survival	159	−1.13145	0.00432	−0.695455	0.50 [0.31–0.80]

### The Association of METTL14 and ZC3H13 Expression With Clinical and Pathological Features

**Figure 5:**
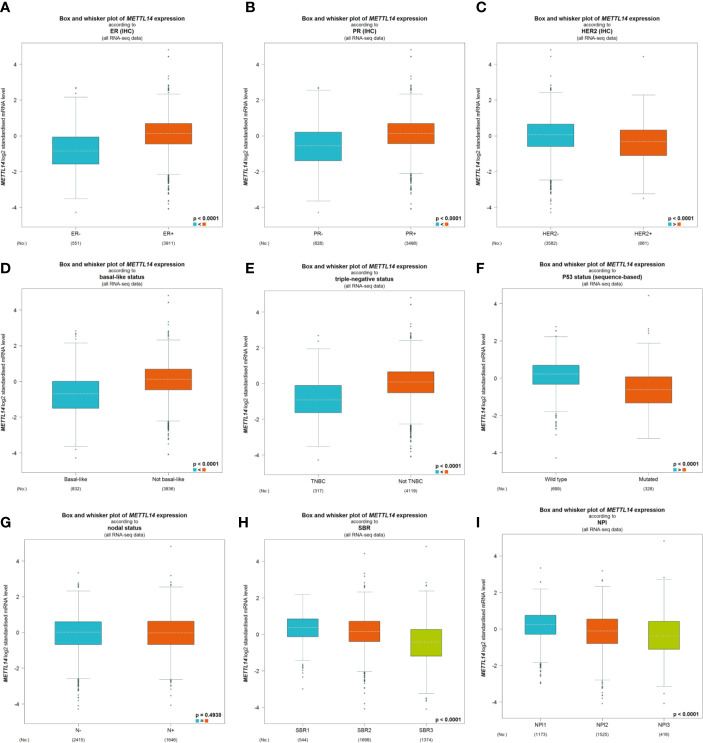
The association of METTL14 expression with clinical and pathological features. Notable global differences between the groups were analyzed by Welch’s t test. **(A)** ER status, estrogen receptor. **(B)** PR status, progesterone receptor. **(C)** HER2 status, human epidermal growth factor receptor 2. **(D)** Basal-like status. **(E)** Triple-negative status. **(F)** P53 status. **(G)** Nodal status. **(H)** SBR status. **(I)** NPI status.

After excluding cases with incomplete clinical data of TCGA breast cancer patients from UCSC Xena, 637 cases were included to analyzed the association of METTL14 and ZC3H13 expression with clinical and pathological features through chi-squared test. The results showed that the expression of METTL14 and ZC3H13 was significantly associated with ER status, PR status, PAM50 subtype. Besides, the proportion of patients with N2–N3 stages is higher in the low expression group of ZC3H13 (**Table 2**). Further, the profiles of 4,712 breast cancer patient cohorts in bc-GenExMiner 4.0 were examined for validation. METTL14 and ZC3H13 expression was significantly decreased in ER-, PR- and basal-like, TNBC groups. However, HER2− patients had somewhat increase in METTL14 and ZC3H13 mRNA expression. In addition, compared with p53 wild type samples, the genes’ mRNA expression was remarkably decreased in p53 mutated samples. As for the Scarff, Bloom and Richardson (SBR) grade status and Nottingham Prognostic Index (NPI) classification, the expression of the two genes gradually decreased with increasing grade and Index (**Figure 5** and **Figure S4**). Generally, with higher rate of SBR or NPI, the lower of the survival rate was associated. There was no significant relationship between nodal status.

**Table 2:** Association between mRNA expression of METTL14, ZC3H13 and clinicopathological characteristics in breast cancer patients (TCGA).

Characteristic	Total	METTL14		P Value	ZC3H13		P Value
		Low expression	High expression		Low expression	High expression	
ER status							
Negative	149	113	36	<0.001	99	50	<0.001
Positive	488	206	282		220	268	
PR status							
Negative	208	142	66	<0.001	128	80	<0.001
Positive	429	177	252		191	238	
HER2 status							
Negative	548	268	280	0.147	267	281	0.089
Positive	89	51	38		52	37	
PAM50 Subtype							
Her2	51	37	14	<0.001	38	13	<0.001
Luminal A	316	120	196		114	202	
Luminal B	140	62	78		83	57	
Basal/Normal	130	100	30		84	46	
T Stage							
T1–T2	547	272	275	0.228	271	276	0.239
T3–T4	90	47	43		48	42	
N Stage							
N0–N1	530	267	263	0.960	252	278	0.009
N2–N3	107	52	55		67	40	
M Stage							
M0	624	311	313	0.760	312	312	0.963
M1	13	8	5		7	6	
Stage							
Stages I–II	490	248	242	0.323	232	258	0.393
Stages III–IV	147	71	76		87	60	

### KEGG and GO Enrichment of Co-Expressed Genes

**Figure 6:**
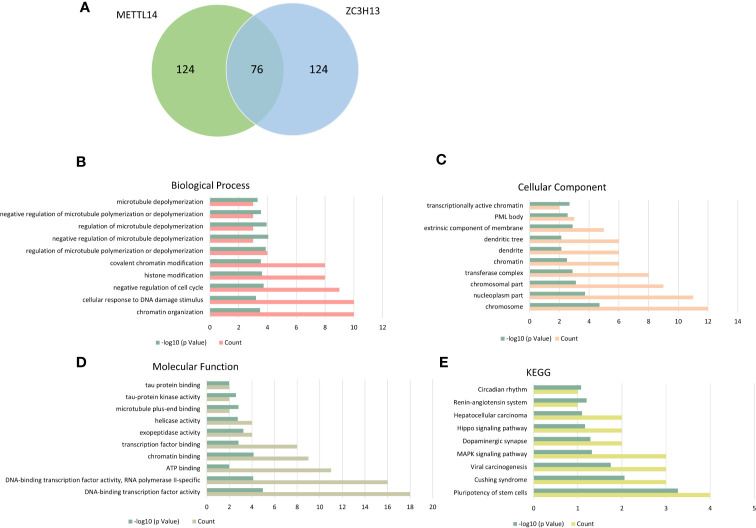
KEGG and GO enrichment of co-expressed genes. **(A)** The top 200 positively co-expressed genes of METTL14 and ZC3H13 in breast cancer (TCGA, PanCancer Atlas) were used as intersection to build a set of 76 common co-expressed genes. **(B–D)** GO enrichment analysis of the 76 common co-expressed genes, including biological process, cellular component and molecular function. **(E)** KEGG pathway enrichment analysis of the 76 common co-expressed genes.

The cBioPortal database was applied to select the top 200 positively co-expressed genes of METTL14 and ZC3H13 based on the data from Breast invasive carcinoma (TCGA, PanCancer Atlas). The co-expressed genes obtained from the two genes were used as intersection to build a set of 76 common co-expressed genes (**Table 3** and **Figure 6A**). Then, we used WebGestalt for functional and pathway enrichment analysis of the 76 co-expressed genes. The results of GO analysis indicated that these genes mainly play a vital role in the regulation of chromatin organization, cellular response to DNA damage stimulus, cell cycle, histone modification, and regulation of microtubule, consistent with their molecular function and cellular component. It has been confirmed that these processes and functions are involved in tumor development and drug sensitivity (**Figures 6B–D**, **Table S1**). Meanwhile, the pathways enriched by KEGG were also related to tumors, including hepatocellular carcinoma, Hippo signaling pathway, MAPK signaling pathway, and pluripotency of stem cells (**Figure 6E**, **Table S1**).

**Table 3:** 76 common co-expressed genes of METTL14 and ZC3H13.

Correlated Gene	METTL14			ZC3H13		
	Spearman’s Correlation	p-Value	q-Value	Spearman’s Correlation	p-Value	q-Value
AFF4	0.664	3.4E-127	5E-124	0.579	3.68E-90	7E-88
ANKRD17	0.564	1.15E-84	9.5E-83	0.545	6.4E-78	5.72E-76
APC	0.649	3.9E-120	3E-117	0.606	1.3E-100	4.49E-98
ARID2	0.612	2.6E-103	7E-101	0.559	7.17E-83	8.62E-81
ARID4A	0.570	8.17E-87	7.7E-85	0.574	3.01E-88	5.11E-86
ASH1L	0.568	4.19E-86	3.7E-84	0.588	1.24E-93	2.95E-91
ATE1	0.620	8.9E-107	3E-104	0.606	1.4E-100	4.86E-98
ATF2	0.573	9.04E-88	9.1E-86	0.592	6.76E-95	1.82E-92
ATG2B	0.599	5.58E-98	1.1E-95	0.575	1.39E-88	2.41E-86
ATRX	0.635	4E-113	2E-110	0.628	4.3E-110	2.8E-107
BDP1	0.601	1.16E-98	2.3E-96	0.563	4.57E-84	6.03E-82
BOD1L1	0.658	2.9E-124	3E-121	0.690	1.4E-141	2.2E-138
CCNT1	0.621	4.9E-107	2E-104	0.609	4.2E-102	1.6E-99
CFAP97	0.678	5.6E-135	1E-131	0.547	1.21E-78	1.13E-76
CHD6	0.598	2.56E-97	4.7E-95	0.550	1.11E-79	1.12E-77
CLCN3	0.694	1.1E-143	3E-140	0.549	3.2E-79	3.06E-77
CLOCK	0.615	2E-104	6E-102	0.583	1.26E-91	2.64E-89
CREBRF	0.640	9.1E-116	6E-113	0.591	1.78E-94	4.66E-92
DDI2	0.585	2.55E-92	3.5E-90	0.602	4.9E-99	1.54E-96
DENND4C	0.594	7.93E-96	1.3E-93	0.571	4.75E-87	7.66E-85
DMXL1	0.649	4.8E-120	4E-117	0.584	9.32E-92	2E-89
DNAJB14	0.737	2.9E-171	1E-167	0.593	1.27E-95	3.51E-93
DPP8	0.612	3E-103	8E-101	0.628	3.3E-110	2.2E-107
ERCC6L2	0.583	1.14E-91	1.5E-89	0.613	1.9E-103	8.6E-101
EXOC6B	0.583	1.83E-91	2.3E-89	0.580	2.74E-90	5.27E-88
GCC2	0.578	7.71E-90	8.7E-88	0.549	2.6E-79	2.52E-77
GIGYF2	0.606	1.4E-100	3.2E-98	0.587	4.51E-93	1.02E-90
GOLGB1	0.577	1.93E-89	2.2E-87	0.544	1.22E-77	1.06E-75
GTF2A1	0.564	1.01E-84	8.4E-83	0.598	2.09E-97	6.01E-95
HECTD1	0.595	2.31E-96	4E-94	0.601	1.93E-98	5.99E-96
IL6ST	0.671	6.3E-131	9E-128	0.548	5.56E-79	5.22E-77
KDM3B	0.623	8.7E-108	3E-105	0.560	3.71E-83	4.54E-81
KIAA1109	0.767	2.1E-193	2E-189	0.621	7.5E-107	4.1E-104
KLHL11	0.663	5.3E-127	7E-124	0.576	9.15E-89	1.63E-86
KLHL28	0.565	5.26E-85	4.4E-83	0.544	9.61E-78	8.48E-76
LATS1	0.571	3.81E-87	3.7E-85	0.623	1E-107	5.8E-105
LCOR	0.635	3.5E-113	2E-110	0.645	5.4E-118	5.2E-115
LMBRD2	0.631	1E-111	5E-109	0.574	2.93E-88	5.01E-86
LNPEP	0.621	5.2E-107	2E-104	0.551	5.13E-80	5.31E-78
MBD5	0.575	1.52E-88	1.6E-86	0.566	2.58E-85	3.6E-83
MINDY2	0.587	2.79E-93	4E-91	0.616	4.6E-105	2.3E-102
N4BP2	0.622	1.6E-107	6E-105	0.608	1.6E-101	5.9E-99
NF1	0.630	4.8E-111	2E-108	0.622	1.5E-107	8.5E-105
PHC3	0.590	3.56E-94	5.4E-92	0.542	5.21E-77	4.42E-75
PIK3C2A	0.602	5.1E-99	1.1E-96	0.583	1.01E-91	2.15E-89
PREPL	0.605	3.5E-100	8E-98	0.542	4.05E-77	3.48E-75
RAPGEF6	0.661	6.1E-126	7E-123	0.563	4.69E-84	6.15E-82
REST	0.624	3.5E-108	1E-105	0.631	2.3E-111	1.7E-108
RNF111	0.630	2.9E-111	1E-108	0.600	2.7E-98	8.25E-96
RSC1A1	0.583	1.85E-91	2.3E-89	0.570	1.46E-86	2.23E-84
SCAF11	0.602	7.2E-99	1.5E-96	0.587	4.41E-93	1.01E-90
SLX4IP	0.615	1.3E-104	4E-102	0.551	5.26E-80	5.42E-78
SMARCAD1	0.692	9.5E-143	2E-139	0.560	4.15E-83	5.04E-81
SPG11	0.615	1.6E-104	5E-102	0.549	2.38E-79	2.32E-77
TAOK1	0.596	1.16E-96	2E-94	0.612	3.7E-103	1.6E-100
TCP11L2	0.569	1.76E-86	1.6E-84	0.561	1.63E-83	2.06E-81
TOGARAM1	0.629	1.1E-110	5E-108	0.569	3.55E-86	5.3E-84
TRIM44	0.570	9.44E-87	8.9E-85	0.559	1.27E-82	1.46E-80
TRIP11	0.591	1.4E-94	2.1E-92	0.547	1.6E-78	1.48E-76
TTBK2	0.594	8.36E-96	1.4E-93	0.643	4.9E-117	4.5E-114
TTC37	0.634	4.9E-113	3E-110	0.549	3.21E-79	3.06E-77
UBR1	0.618	7.9E-106	2E-103	0.557	6.42E-82	7.33E-80
UHMK1	0.619	2.3E-106	8E-104	0.566	3.95E-85	5.42E-83
USP8	0.604	7.9E-100	1.8E-97	0.552	2.81E-80	2.95E-78
UTP14C	0.576	5.42E-89	5.8E-87	0.817	4.5E-239	4.6E-235
WDFY3	0.701	4.5E-148	1E-144	0.619	2.9E-106	1.5E-103
ZBTB37	0.575	2.21E-88	2.3E-86	0.551	6.72E-80	6.85E-78
ZKSCAN1	0.591	1.2E-94	1.9E-92	0.580	1.85E-90	3.59E-88
ZKSCAN8	0.569	1.77E-86	1.6E-84	0.616	9.8E-105	4.6E-102
ZNF510	0.602	4.1E-99	8.6E-97	0.570	7.73E-87	1.22E-84
ZNF585B	0.577	2.94E-89	3.2E-87	0.560	3.24E-83	4.01E-81
ZNF619	0.624	1.7E-108	7E-106	0.564	1.29E-84	1.75E-82
ZNF678	0.583	1.68E-91	2.1E-89	0.579	5.67E-90	1.07E-87
ZNF791	0.571	6.52E-87	6.2E-85	0.587	3.26E-93	7.55E-91
ZNF81	0.588	1.34E-93	2E-91	0.653	8.6E-122	8.7E-119
ZNF827	0.602	4E-99	8.6E-97	0.570	9.62E-87	1.49E-84

### METTL14 and ZC3H13 Associated PPI Network Construction

**Figure 7:**
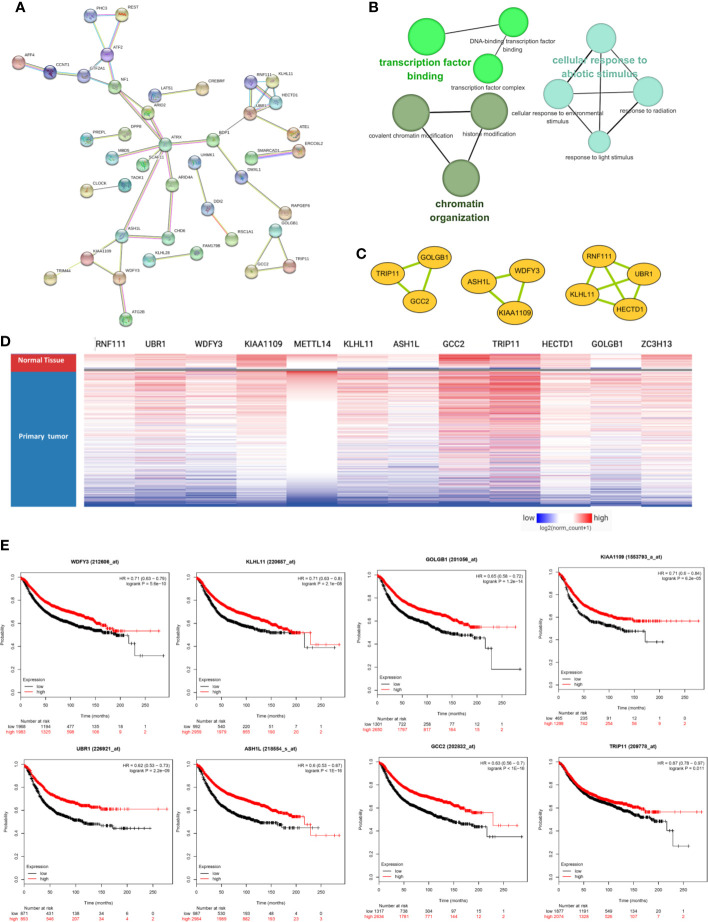
METTL14 and ZC3H13 associated PPI network construction. **(A)** PPI network of the 76 co-expressed genes by the STRING database. **(B)** The function analysis of this PPI network. **(C)** Three crucial modules selected by Cytoscape from the PPI network. **(D)** The clustered heatmap of the 10 genes in crucial modules and METTL14, ZC3H13 in TCGA BRCA by UCSC Xena. **(E)** Prognostic significance of hub genes expression in breast cancer patients based on the Kaplan–Meier plotter database.

The PPI network was constructed based on the 76 co-expressed genes by using the STRING database, and the crucial modules were built by Cytoscape (**Figure 7A**). The function of this PPI network is mainly involved in transcription factor binding, chromatin organization, and cellular response to abiotic stimulus (**Figure 7B**). There were 10 genes in three crucial modules, including GOLGB1, TRIP11, GCC2, ASH1L, WDFY3, KIAA1109, RNF111, KLHL11, HECTD1, and UBR1 (**Figure 7C**). The clustered heatmap of these 10 genes and METTL14, ZC3H13 was analyzed and visualized by UCSC Xena, indicating their related expression pattern (**Figure 7D**, **Figure S6**). Furthermore, the prognostic roles of the 10 genes were performed using Kaplan–Meier plotter. Accordingly, WDFY3, KLHL11, GOLGB1, KIAA1109, UBR1, ASH1L, GCC2, and TRIP11 exhibited poor RFS in the lower expression groups (**Figure 7E**). In TCGA breast cancer samples, only reduced expression of GCC2 and ASH1L could show poor RFS (**Figure S5**).

### Co-Expression of APC and METTL14, ZC3H13

**Figure 8:**
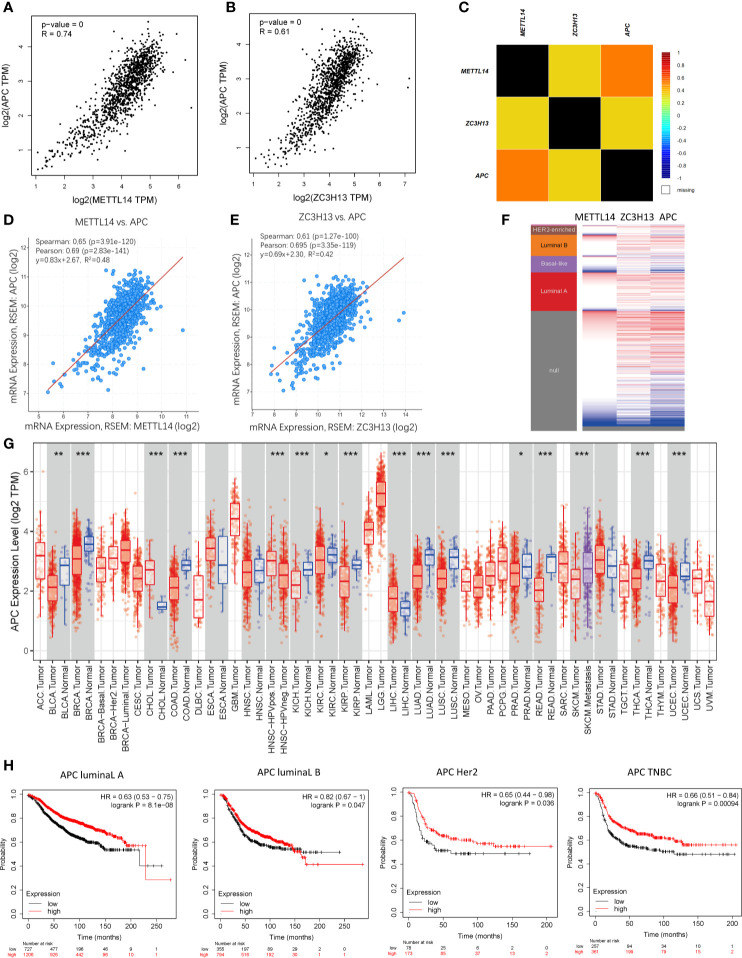
Co-expression of APC and METTL14, ZC3H13. **(A, B)** The correlation between APC and METTL14, ZC3H13 mRNA expression determined using GEPIA. **(C)** The relationship between APC and METTL14, ZC3H13 in breast cancer analyzed using bc-GenExMiner v4.0. **(D, E)** The correlation between APC and METTL14, ZC3H13 mRNA expression *via* the cBioPortal online database (TCGA, PanCancer Atlas). **(F)** Heat map of APC expression and METTL14, ZC3H13 mRNA expression across PAM50 breast cancer subtypes in the TCGA using UCSC Xena. **(G)** The expression of APC in cancer tissues and normal control in TCGA database generated by TIMER database. **(F)** The RFS curves of APC gene expression in four types breast cancer patients, including luminal type A, luminal type B, HER2 enriched type and triple negative type (TNBC). ***P < 0.001, **P < 0.01, *P < 0.05.

Based on the enrichment results of KEGG pathway (**Table S1**), we noticed a familiar tumor suppressor gene, APC, which is associated with several carcinoma related pathways: Hippo signaling pathway, MAPK signaling pathway, and pluripotency of stem cells. It is confirmed that APC acts as an antagonist of the Wnt signaling pathway in several cancer types, and the low expression of APC promotes tumor progression. Besides, it is also involved in other processes, like cell migration and adhesion, transcriptional activation, and apoptosis (29, 30). The data and results from different databases and datasets showed that both METTL14 and ZC3H13 had high correlation coefficients with APC (R > 0.6, P < 0.05) (**Figures 8A–F**). Then, a thorough inquiry of the expression profile of APC was made using TIMER database, indicating that its mRNA expression was significantly decreased in enriched cancer types, such as breast cancer, colorectal cancer, lung squamous cell carcinoma, lung adenocarcinoma, *etc* (**Figure 8G**). Subsequently, the prognostic value of APC (Prob ID: 215310 at) in breast cancer was also performed using Kaplan–Meier plotter; the results confirmed that the lower expression of APC mRNA was associated with the worse RFS of different breast cancer types (**Figure 8H**). However, there was no relationship between the expression of APC and RFS in TCGA breast cancer samples (**Figure S5**).

### Correlation of METTL14 and ZC3H13 Expression With Seven Types of Immune Cells

**Figure 9:**
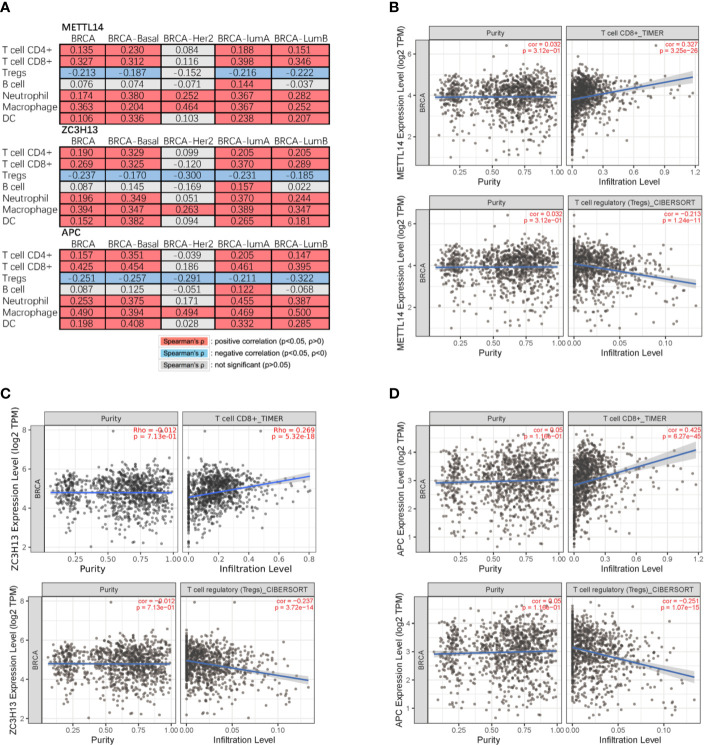
Correlation of METTL14 and ZC3H13 expression with seven types of immune cells. **(A)** Spearman’ s correlation of METTL14 and ZC3H13 expression with 7 types of immune cells in breast cancer patients. **(B)** The correlation between METTL14 expression level and infiltrating CD8+ T cells, Treg cells in breast cancer. **(C)** The correlation between ZC3H13 expression level and infiltrating CD8+ T cells, Treg cells in breast cancer. **(D)** The correlation between APC expression level and infiltrating CD8+ T cells, Treg cells in breast cancer.

Studies have confirmed that the excessive activation of the Wnt signaling pathway in tumors can lead to tumor immunosuppression and immune escape, further promoting tumor invasion and metastasis (31, 32). Based on the correlation between METTL14, ZC3H13, and APC expression pattern, we speculated that METTL14 and ZC3H13 would also be indirectly involved in mediating the immune response of tumors. So, we analyzed the correlation between the genes expression and seven types of infiltrating immune cells (CD4+ T cells, CD8+ T cells, Treg cells, B cells, neutrophils, macrophages and dendritic cells) by TIMER database. METTL14, ZC3H13, and APC were all significantly positively correlated with CD4+ T cells, CD8+ T cells, neutrophils, macrophages, and dendritic cells in BRCA, negatively with Treg cells (P < 0.05) (**Figure 9A**). In different breast cancer subtypes, the correlations were not all the same. Importantly, APC showed a better correlation with these immune cells than METTL14 and ZC3H13 (**Figures 9B–D**). Besides, we also calculated the association between immune infiltrates and somatic CNV of METTL14 and ZC3H13 in breast cancer, and these immune cells showed diverse enrichment trends in different CNV types across different types of breast cancer (**Figure S7**).

### Validation of METTL14 and ZC3H13 Expression in Breast Cancer Tissues

**Figure 10:**
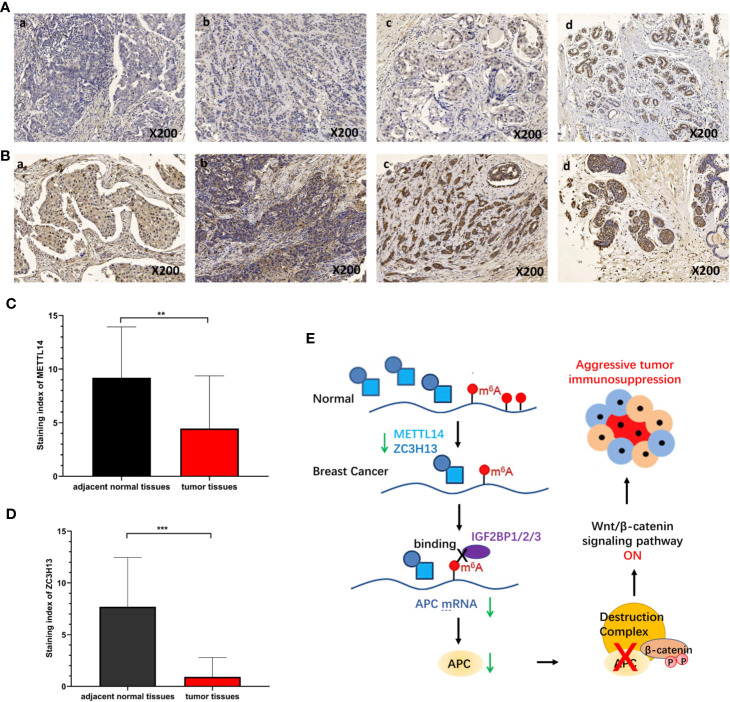
Validation of METTL14 and ZC3H13 expression in breast cancer tissues. **(A)** Representative immunohistochemical (IHC) staining showing METTL14 expression in breast tumor and adjacent normal tissues (a: tumor tissue, nucleus−; b: tumor tissue, nucleus+; c: tumor tissue, nucleus++; d: adjacent normal tissue, nucleus+++). **(B)** Representative immunohistochemical (IHC) staining showing ZCH313 expression in breast tumor and adjacent normal tissues (a: tumor tissue, nucleus−, cytoplasm−; b: tumor tissue, nucleus+, cytoplasm+; c: tumor tissue, nucleus++, cytoplasm+; d: adjacent normal tissue, nucleus+++, cytoplasm+). **(C, D)** Staining index of METTL14 and ZC3H13 in nucleus in breast tumor and adjacent normal tissues. **(E)** Scientific hypothesis of METTL14/ZC3H13-m6A-APC/Wnt signaling pathway. ***P < 0.001, **P < 0.01.

To verify the results of bioinformatics analysis, immunohistochemical (IHC) staining was performed on tissue microarray slides containing 50 breast cancer tissues and 10 adjacent normal tissues. The staining patterns of METTL14 and ZC3H13 in tumor and normal tissues were shown in **Figures 10A, B**. METTL14 was mainly localized in the nucleus but not in other parts of cells, and ZCH3H13 was observed not only in the nucleus but also in the cytoplasm in cells. Considering that m6A modification took place in the nucleus, we compared the expression level of the two proteins in the nucleus. The levels of the expression were quantitated by the Staining index based on tumor cell proportion and staining intensity. The result further confirmed that both METTL14 and ZC3H13 were significantly down-regulated in the nucleus in tumor tissues relative to normal control, which was consistent with the results of bioinformatics analysis on RNA levels (**Figures 10C, D**).

## Discussion

The pathological and biological functions of enriched chemical modifications of proteins and DNA have been well verified and extensively studied. Recently, m6A modification, as a new layer of RNA epigenetic regulation, has also been increasingly studied in human diseases including cancer (33). With the development of m6A sequence technology, m6A modification sites (RRACH, R = A or G; H = A, C, or U) which are mainly enriched near stop codons, in 3′-untranslated regions (3′-UTRs) and within long internal exons have been identified in a great many of coding or non-coding RNAs (34). With the in-depth study of the process of m6A, researchers have identified a series of proteins related to this modification, including “writers” (METTL3, METTL14, WTAP, RBM15, RBM15B, and ZC3H13), “erasers” (FTO and ALKBH5) and “readers” (IGF2BP1/2/3, YTHDF1/2/3, YTHDC2, HNRNPA2B1, *etc*) (7). By regulating the level of m6A modification, the abnormal expression of these proteins in tumors affects the fate and metabolism of tumor-related mRNAs, including translation, splicing, translocation, degradation, and processing and plays an important role in the occurrence and development of cancer (35). For the m6A “writers”, several previous studies have demonstrated that dysregulation of METTL3 and METTL14 could contribute to human carcinogenesis. METTL3 was up-regulated in acute myeloid leukemia cells, enhanced translation of c-MYC, BCL2, and PTEN mRNAs, blocks cell differentiation and apoptosis, and promotes leukemia progression (36, 37). METTL3 was also over-expressed in pancreatic cancer, bladder cancer, glioma and gastric cancers, and promotes proliferation, invasion, and drug resistance of cancer cells (38–41). By contrast, METTL14 was down-regulated in colorectal cancer and hepatocellular carcinoma and could inhibit cancer cells’ growth and metastasis by regulating m6A-dependent primary microRNA processing (12, 42).

In agreement with previous reports (12, 42), our study also identified METTL14 as a tumor suppressor gene in breast cancer, as well as ZC3H13, while other m6A “writers” (METTL3, RBM15, and RBM15B) were not dysregulated in tumor tissues compared with normal control based on the data from TCGA and Oncomine databases. We also compared their expressions in tissue samples, and the obtained expression was consistent with the bioinformatics analysis. What is more, survival analysis showed that low METTL14 and ZC3H13 expression could demonstrate poor prognosis in breast cancer for OS and RFS. Low expression of METTL14 and ZC3H13 was related to breast cancer progression (increased SBR grade status and NPI classification) and was significantly decreased in ER-, PR- and basal-like, TNBC patients. The above results suggested that the down-regulated METTL14 and ZC3H13 led to a decrease in m6A modification levels in breast cancer, which promoted cancer cells’ invasion and metastasis. Interestingly, it has been demonstrated that m6A “eraser” FTO was significantly up-regulated in breast cancer, which could promote breast cancer cell proliferation, colony formation and reduce apoptosis (43). In addition, it is confirmed that ALKBH5 was also up-regulated in breast carcinoma, promoted demethylation of m6A, and then improved the stemness of breast cancer cells (44). In summary, these studies combined with our results have confirmed the reduction of m6A modification levels in breast cancer.

M6A “writers” and “erasers” determine the m6A modification on a specific mRNA. It is also important that m6A “readers” can specifically recognize and bind the modification sites to sort the mRNA and transmit information, thereby establishing an effective m6A associated network (7). To further elucidate the downstream molecular mechanism involved in METTL14 and ZC3H13 by regulating m6A in breast cancer, we selected a set of 76 common positively co-expressed genes of METTL14 and ZC3H13. The pathways enriched by KEGG of these genes were associated with tumors, such as hepatocellular carcinoma, Hippo signaling pathway, MAPK signaling pathway and pluripotency of stem cells. Importantly, we noticed a familiar tumor suppressor gene, APC, among these genes. It was confirmed that APC could inhibit the Wnt signaling pathway, a carcinogenic signaling pathway in several cancers, and the low expression of APC promoted tumor progression (45). Afterwards, we analyzed and confirmed that there were multiple m6A sites on the APC mRNA *via* the Whistle database (**Table S2**). In addition, by analyzing RNA binding proteins that can bind to APC mRNA by Starbase database, we found that m6A “reader” Insulin Like Growth Factor 2 MRNA Binding Protein 1/2/3 (IGF2BP1/2/3) could bind to several regions of APC mRNA (**Figure S8**). IGF2BP1/2/3 can recognize and bind the conserved GG(m6A)C sequence on mRNA under normal and stress conditions and enhance the stability of mRNA. In HEK293T cells, IGF2BP1/2/3 mainly recognizes and binds to the m6A sites near the stop codon of transcription, leading to the accumulation of carcinogenic products such as MYC (46). In colorectal cancer, IGF2BP2 can recognize m6A sites in the CDS region of SOX2, enhancing its stability and preventing its degradation, leading to the occurrence and development of colorectal cancer (47). Based on these documents and results, we proposed a scientific hypothesis (**Figure 10E**): METTL14 and ZC3H13 can promote the m6A modification process and level of APC mRNA, after which IGF2BP1/2/3 recognizes the m6A sites and improves the stability of APC mRNA. However, since the expressions of METTL14 and ZC3H13 in breast cancer were significantly reduced, this process is reversed, resulting in a decrease in the stability of APC mRNA and inhibiting the protein level of APC. Thus, a decrease in APC protein level leads to abnormal activation of Wnt signaling pathway in breast cancer.

Abnormal activation of the Wnt pathway is thought to positively regulate various properties of tumor cells, including proliferation, survival, and metastasis (48). In addition, Wnt signaling also plays an important role in the regulation of tumor immune microenvironment, which can regulate the infiltration and activity of various types of T cells in tumors. Usually excessive activation of Wnt signaling pathway in tumors can lead to tumor immunosuppression and immune escape (49, 50). In colorectal cancer, inhibiting the activation of the Wnt signaling pathway can reduce the infiltration of Treg cells in the tumor and up-regulate the content of CD8+ T cells in the tumor, thereby enhancing the response to immunotherapy (51). Based on the correlation of APC and METTL14, ZC3H13 in breast cancer, we speculated that METTL14 and ZC3H13 were indirectly involved in mediating tumor immune responses. Analysis of the correlation between gene expression and seven infiltrating immune cells *via* the TIMER database showed that METTL14, ZC3H13, and APC were all significantly positively correlated with CD4+ T cells, CD8+ T cells, neutrophils, macrophages, and dendritic cells in BRCA, negatively with Treg cells. This indicated that low expression of METTL14 and ZC3H13 promoted immunosuppression in the breast cancer, which is also an important downstream mechanism for their role in the progression and metastasis of breast cancer.

In conclusion, our study revealed that METTL14 and ZC3H13, as two tumor suppressor genes, were down-regulated in breast cancer, and the low expression of METTL14 and ZC3H13 was negatively correlated with the OS and RFS. Their abnormal expression promoted the development of breast cancer by affecting pathways related to tumor development. Among one of these downstream molecular mechanisms, they could affect the m6A modification level of APC mRNA, then activate the Wnt signaling pathway. However, further experimental studies are necessary to provide solid confirmation of our scientific hypothesis and results.

## Data Availability Statement

The original contributions presented in the study are included in the article/**Supplementary Material**. Further inquiries can be directed to the corresponding author.

## Author Contributions

P-JG and J-WZ contributed to the conception of the study. P-JG and Y-CS contributed to experimental technology and experimental design. P-JG, YY, XH, and W-JS performed the data analyses. P-JG, S-RH, and Y-FZ wrote the manuscript. LW and J-WZ supervised the study. All authors contributed to the article and approved the submitted version.

## Funding

This work was supported by funds from Health commission of Hubei Province scientific research project (WJ2019H020, WJ2019H028).

## Conflict of Interest

The authors declare that the research was conducted in the absence of any commercial or financial relationships that could be construed as a potential conflict of interest.

## Supplementary Material

Supplementary Table 1 Pathway and GO enrichment results of 76 common co-expressed genes of METTL14 and ZC3H13.

Supplementary Table 2 m6A sites on APC mRNA (Whistle database).

Figure S1 The expression of WTAP in breast cancer tissues vs normal control in the datasets of Oncomine databas.

Figure S2 The expression of METTL3 and RBM15B in cancer tissues and normal control in TCGA database generated by TIMER database. * * *P < 0.001, * * P < 0.01, * P < 0.05.

Figure S3 Prognostic roles of METTL14 and ZC3H13 gene expression in TCGA breast cancer patients. **(A, B)** The OS curves of METTL14 and ZC3H13 gene expression. **(C, D)** The RFS curves of METTL14 and ZC3H13 gene expression. RFS, relapse-free survival; OS, overall survival.

Figure S4 The association of ZC3H13 expression with Clinical and pathological features. Notable global differences between the groups were analyzed by Welch’s t test. **(A)** ER status, estrogen receptor. **(B)** PR status, progesterone receptor. **(C)** HER2 status, human epidermal growth factor receptor 2. **(D)** Basal-like status. **(E)** Triple-negative status. **(F)** P53 status. **(G)** Nodal status. **(H)** SBR status. **(I)** NPI status.

Figure S5 The RFS curves of the 10 genes in crucial modules and APC in TCGA breast cancer samples. RFS, relapse-free survival.

Figure S6 The correlation of the 10 genes in crucial modules and METTL14, ZC3H13 in TCGA breast cancer samples.

Figure S7 The association between immune infiltrates and somatic CNV of METTL14 and ZC3H13 in breast cancer.

Figure S8 The IGF2BP1/2/3 –APC interaction supported by CLIP-seq Data (Starbase database).

## Abbreviations

m6A, N6-methyladenosine; METTL3, methyltransferase-like 3; METTL14, methyltransferase-like 14; WTAP, Wilms tumor 1 associated protein; ZC3H13, Zinc Finger CCCH-Type Containing 13; FTO, obesity-associated protein; ALKBH5, alkB homolog 5; APC, Adenomatous polyposis coli; TCGA, The Cancer Genome Atlas; BRCA, Breast invasive carcinoma; GO, Gene Ontology; KEGG, Kyoto Encyclopedia of Genes and Genomes; BP, biological processes; CC, cellular components; MF, molecular functions; SBR grade, Scarff, Bloom and Richardson grade; NPI, Nottingham Prognostic Index; HR, hazard ratio; CI, Confidence interval. ER, estrogen receptor; PR, progesterone receptor; HER2, human epidermal growth factor receptor 2.
