# Supplementary material for: Analysis of N6-Methyladenosine Methyltransferase Reveals METTL14 and ZC3H13 as Tumor Suppressor Genes in Breast Cancer
Source: Front Oncol. 2020 Dec 9;10:578963. doi: 10.3389/fonc.2020.578963 (PMC7757663; doi:10.3389/fonc.2020.578963)
Supplement: Supplementary Table 2 — m6A sites on APC mRNA (Whistle database). [file Table_2.docx]

| geneName | mod_name | chromosome | modStart | modEnd | width | strand |
| --- | --- | --- | --- | --- | --- | --- |
| APC | m6A_2058 | chr5 | 112179214 | 112179215 | 1 | + |
| APC | m6A_5055 | chr5 | 112176649 | 112176650 | 1 | + |
| APC | m6A_6702 | chr5 | 112179068 | 112179069 | 1 | + |
| APC | m6A_8662 | chr5 | 112175956 | 112175957 | 1 | + |
| APC | m6A_8685 | chr5 | 112179634 | 112179635 | 1 | + |
| APC | m6A_9769 | chr5 | 112173604 | 112173605 | 1 | + |


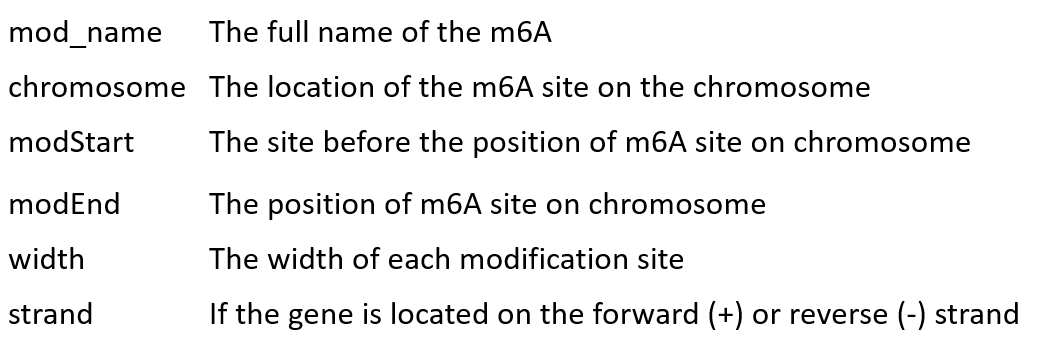
Table S2. m6A sites on APC mRNA (Whistle database)
