# Supplementary material for: Analysis of N6-Methyladenosine Methyltransferase Reveals METTL14 and ZC3H13 as Tumor Suppressor Genes in Breast Cancer
Source: Front Oncol. 2020 Dec 9;10:578963. doi: 10.3389/fonc.2020.578963 (PMC7757663; doi:10.3389/fonc.2020.578963)
Supplement: Figure S1 — The expression of WTAP in breast cancer tissues vs normal control in the datasets of Oncomine databas. [file Presentation_1.pptx]

## Slide 1
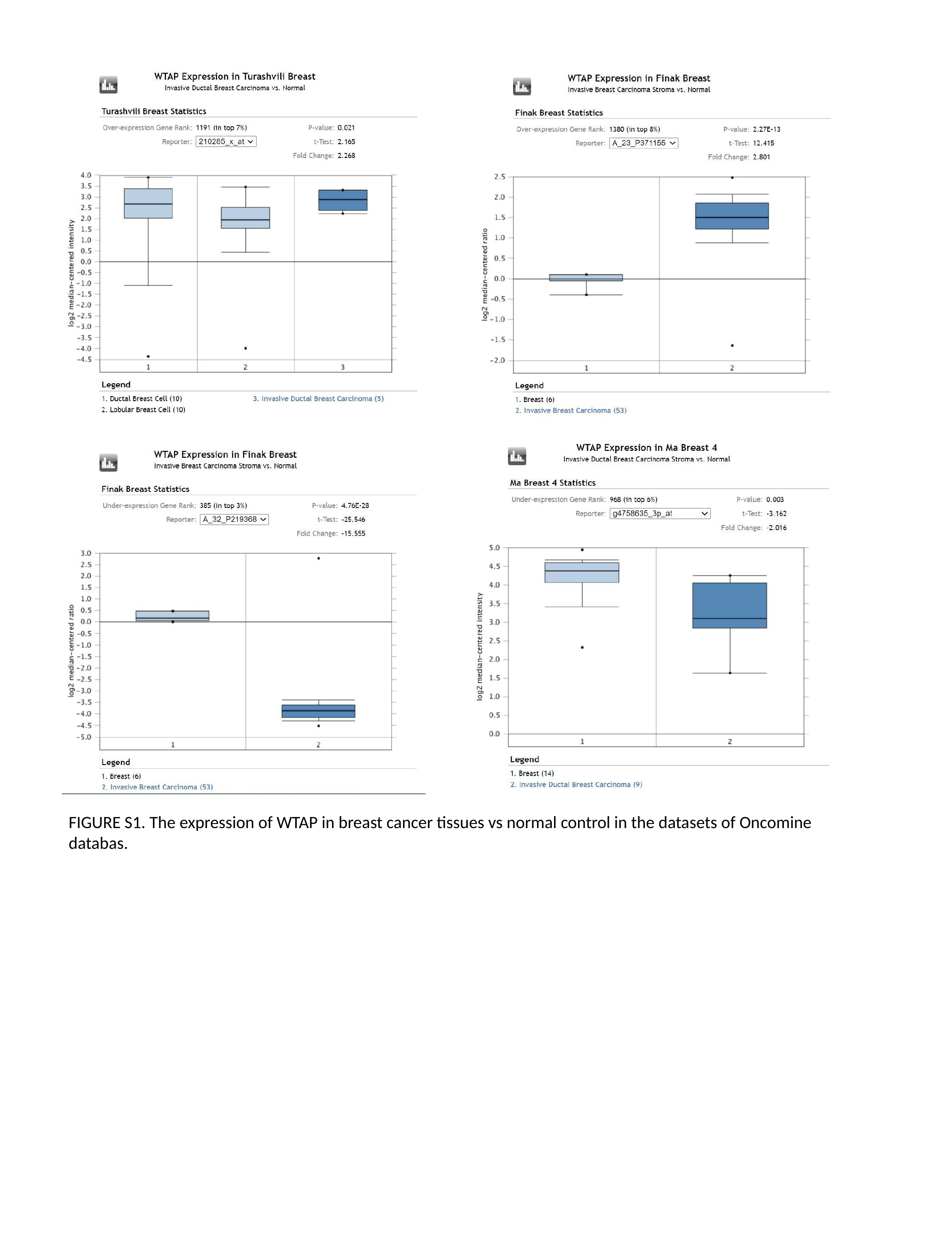

FIGURE S1. The expression of WTAP in breast cancer tissues vs normal control in the datasets of Oncomine databas.

## Slide 2
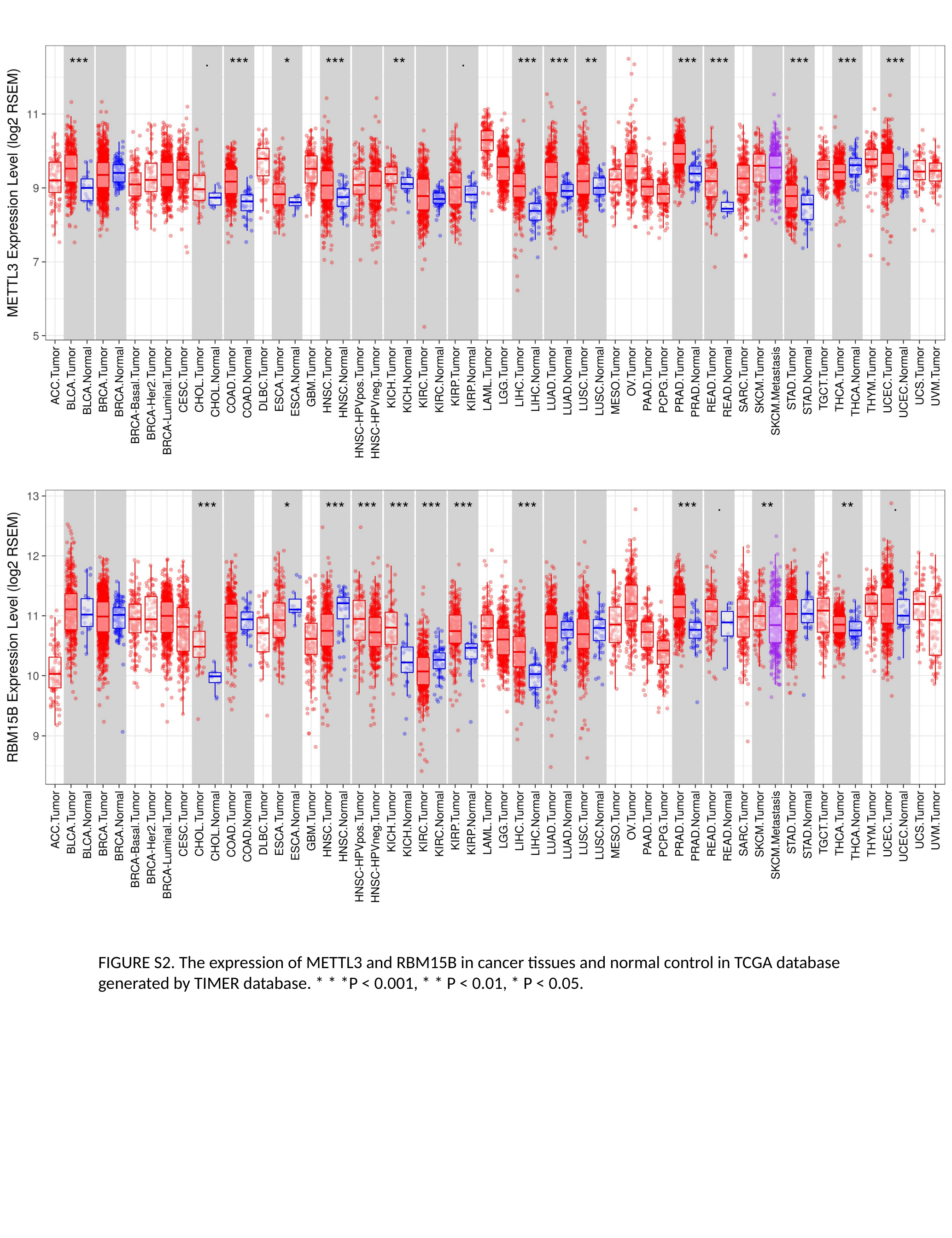

FIGURE S2. The expression of METTL3 and RBM15B in cancer tissues and normal control in TCGA database generated by TIMER database. * * *P < 0.001, * * P < 0.01, * P < 0.05.

## Slide 3
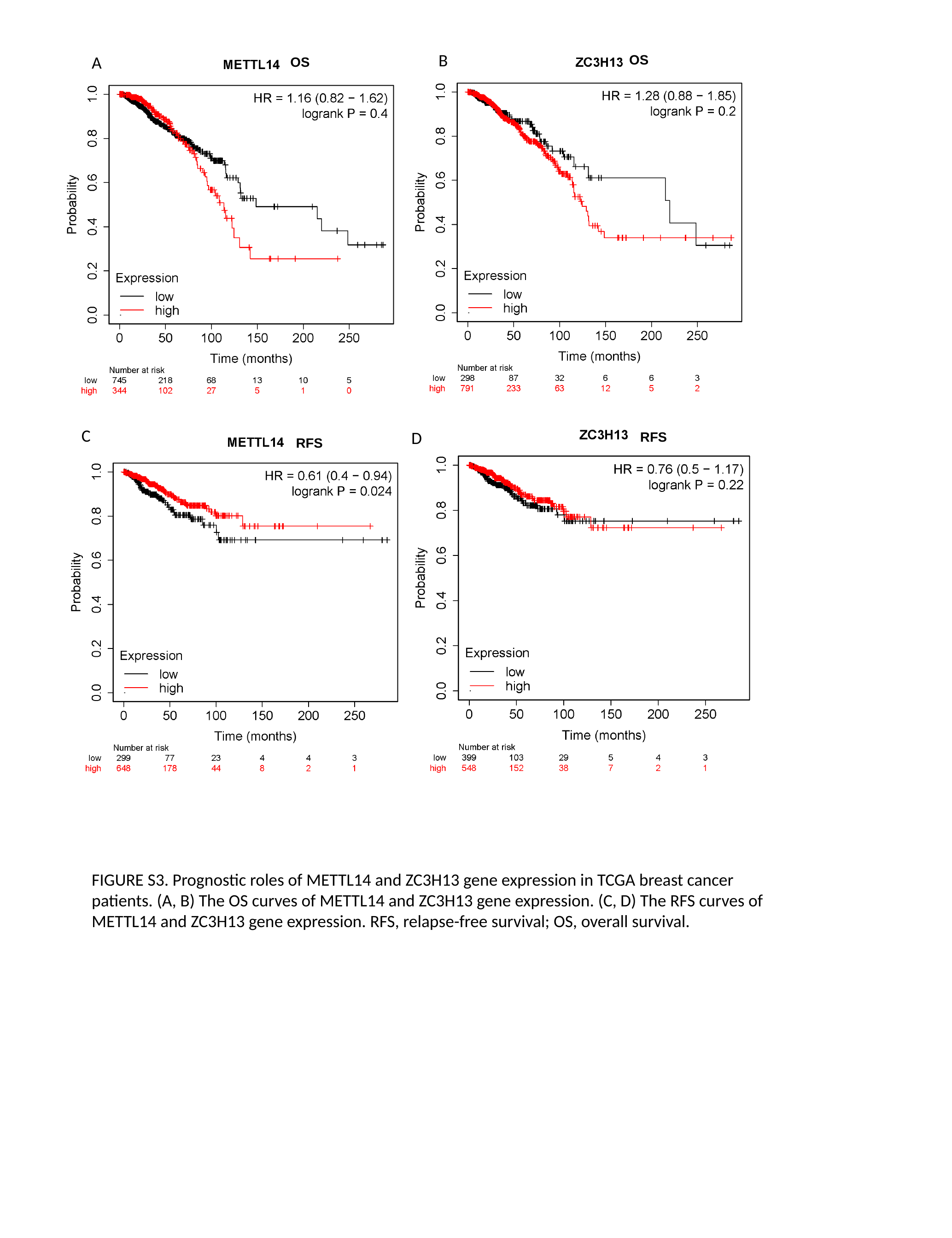

B
A
OS
OS
RFS
RFS
C
D
FIGURE S3. Prognostic roles of METTL14 and ZC3H13 gene expression in TCGA breast cancer patients. (A, B) The OS curves of METTL14 and ZC3H13 gene expression. (C, D) The RFS curves of METTL14 and ZC3H13 gene expression. RFS, relapse-free survival; OS, overall survival.

## Slide 4
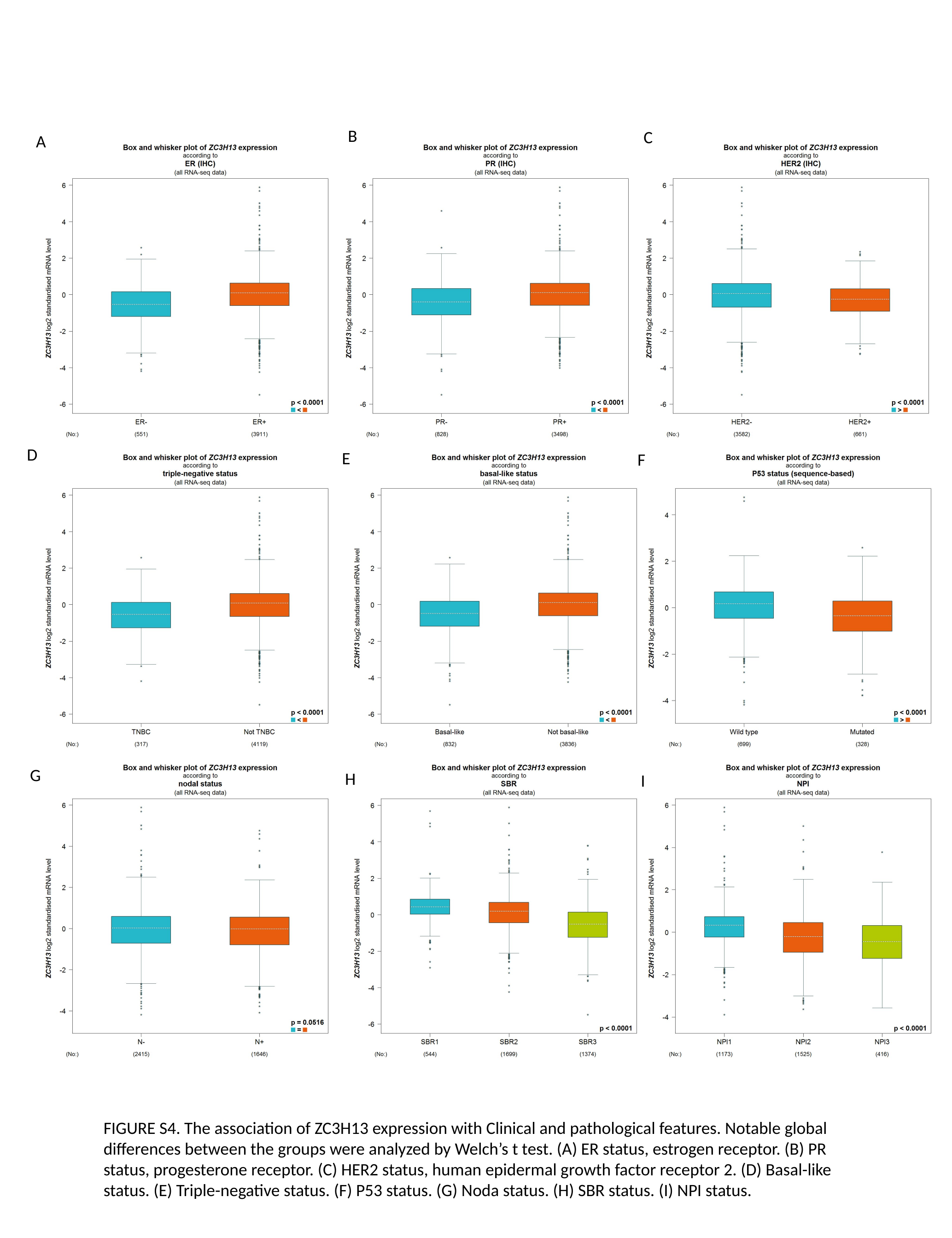

B
C
A
D
E
F
G
H
I
FIGURE S4. The association of ZC3H13 expression with Clinical and pathological features. Notable global differences between the groups were analyzed by Welch’s t test. (A) ER status, estrogen receptor. (B) PR status, progesterone receptor. (C) HER2 status, human epidermal growth factor receptor 2. (D) Basal-like status. (E) Triple-negative status. (F) P53 status. (G) Noda status. (H) SBR status. (I) NPI status.

## Slide 5
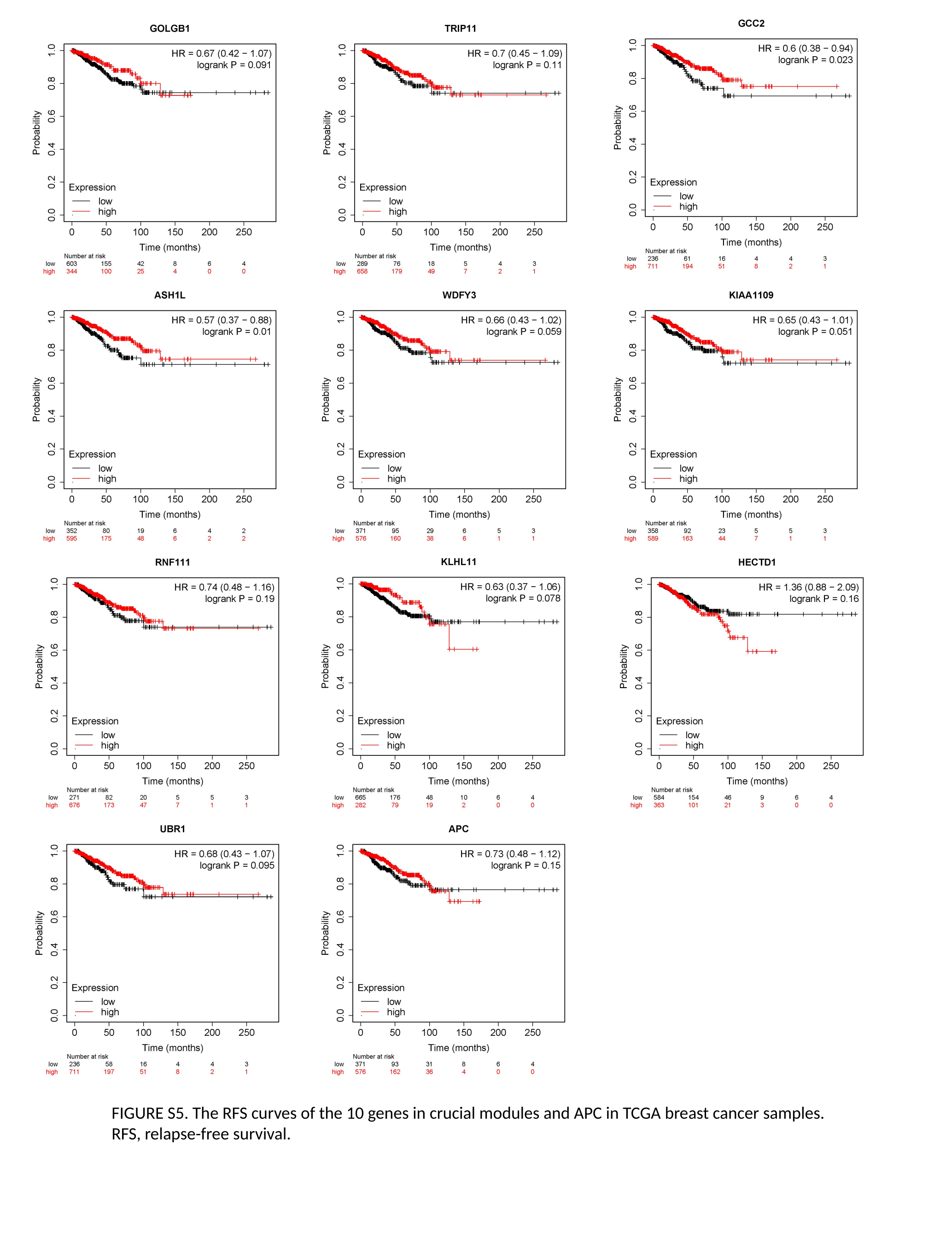

FIGURE S5. The RFS curves of the 10 genes in crucial modules and APC in TCGA breast cancer samples.
RFS, relapse-free survival.

## Slide 6
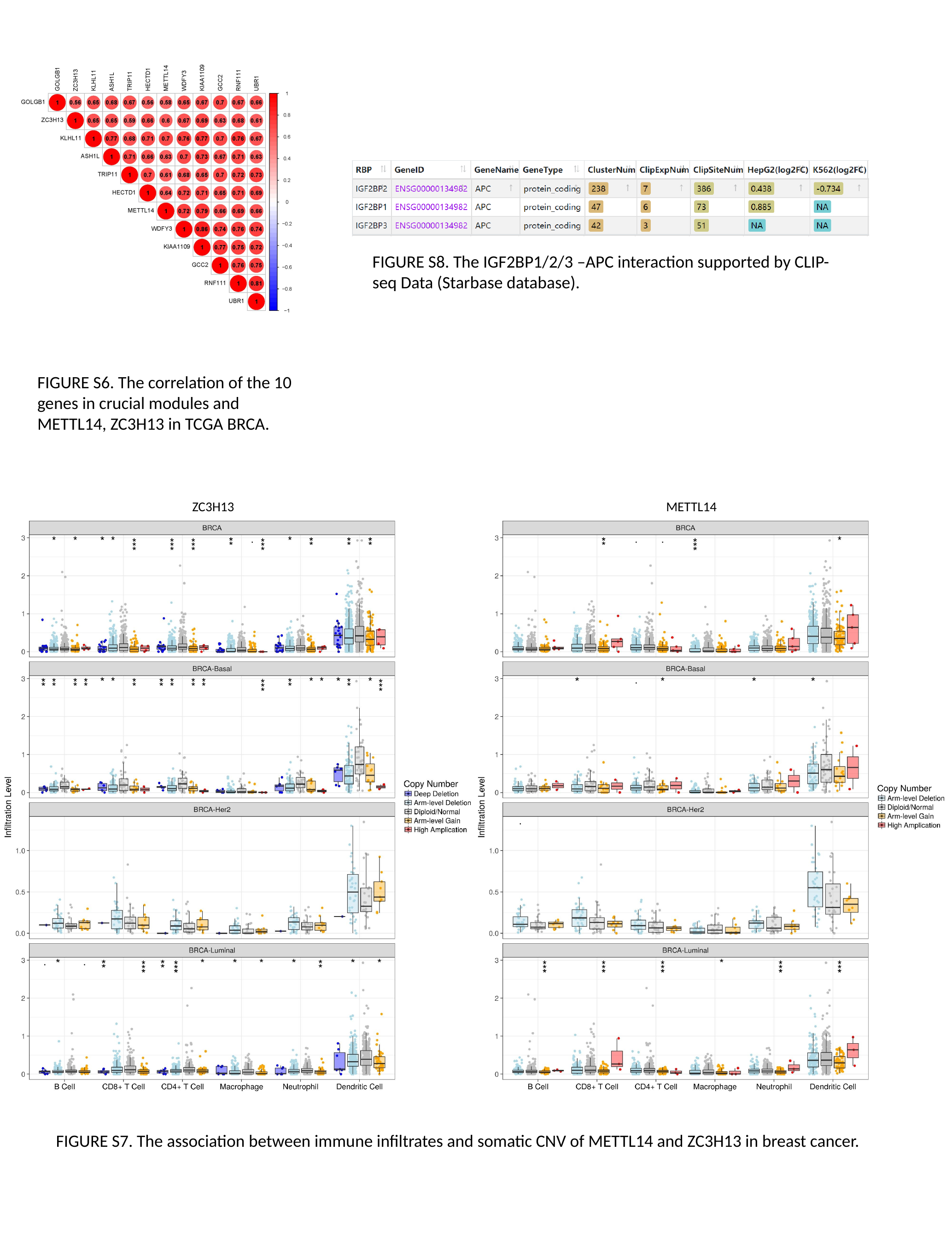

FIGURE S8. The IGF2BP1/2/3 –APC interaction supported by CLIP-seq Data (Starbase database).
FIGURE S6. The correlation of the 10 genes in crucial modules and METTL14, ZC3H13 in TCGA BRCA.
ZC3H13
METTL14
 FIGURE S7. The association between immune infiltrates and somatic CNV of METTL14 and ZC3H13 in breast cancer.
